# Use of low-dose combined therapy with gemcitabine and paclitaxel for advanced urothelial cancer patients with resistance to cisplatin-containing therapy: a retrospective analysis

**DOI:** 10.1007/s00280-012-1938-3

**Published:** 2012-08-03

**Authors:** Yasuyoshi Miyata, Koichiro Nomata, Kojiro Ohba, Tomohiro Matsuo, Yuji Sagara, Hiroshi Kanetake, Hideki Sakai

**Affiliations:** Department of Nephro-Urology, Nagasaki University Graduate School of Biomedical Science, 1-7-1 Sakamoto, Nagasaki, 852-8501 Japan

**Keywords:** Urothelial cancer, Gemcitabine, Paclitaxel, Toxicity, Pain relief

## Abstract

**Purpose:**

The prognosis of patients with advanced and recurrent urothelial cancer (UC) is poor. Although cisplatin (CDDP)-containing chemotherapy is the most effective regimen in these patients, there is no other established chemotherapeutic regimen. We administered combination therapy with low-dose gemcitabine (GEM) and paclitaxel (PTX), named low-dose gemcitabine–paclitaxel (LD-GP) therapy, as salvage therapy for these patients. The aim was to evaluate the anti-tumoral effects, relief of pain, and toxicity of LD-GP therapy in patients with resistance to CDDP-containing therapy.

**Patients and methods:**

Thirty-five patients with advanced UC, previously treated with CDDP-containing regimens, were treated with LD-GP therapy (GEM, 700 mg/m^2^ + PTX, 70 mg/m^2^ on day 1 and 8, repeated every 28 days). Pain was measured on a visual analog scale before and after treatment. Pain relief and survival were compared between this and other treatment regimens.

**Results:**

None of the patients had complete response to LD-GP therapy. Partial response and stable disease were seen in 25.7 and 62.9 % of patients, respectively. Kaplan–Meier curves showed better survival in patients with LD-GP therapy than with others (*p* = 0.034). Twenty-eight patients (80.0 %) had adequate pain relief, and only two patients needed to increase their analgesics. Other regimens demonstrated pain relief in 30.4 % of patients. Common toxicities included leukopenia, with five patients requiring granular colony-stimulating factor therapy (14.3 %). The most common non-hematologic toxicity was fatigue (*n* = 7, 17.1 %).

**Conclusions:**

LD-GP therapy is feasible and well tolerated as salvage therapy in patients with advanced UC with resistance to CDDP-containing therapy.

## Introduction

Urothelial cancer (UC) has a high prevalence rate among the elderly. Almost all UC patients with low-grade and low-stage disease can be cured by definitive local therapy, including transurethral resection (TUR). On the other hand, unfortunately, nearly two-thirds of those with muscle invasion subsequently show regional or systemic disease recurrence. The prognosis of patients with unresectable and metastatic UC is poor, with an average survival rate in untreated patients of 3–6 months [1]. In addition, patients with recurrence after radical cystectomy showed a 1- and 3-year survival of only 17 and 6 %, respectively [2]. Currently, systemic chemotherapy is the only therapeutic modality that produces somewhat long-term survival in these patients.

Cisplatin (CDDP) is one of the most commonly used chemotherapeutic agents for patients with UC. Combined chemotherapy with methotrexate, vinblastine, doxorubicin, and CDDP (MVAC) has been the most common and useful regimen for advanced UC since the 1980 s. However, a large trial with long-term follow-up on MVAC showed a progression-free survival rate at 6 years of only 3.7 % [3]. Furthermore, administration of MVAC to elderly patients presents many and varied problems and considerable toxicity, including myelosuppression, nephrotoxicity, and neuropathy. Recently, the combination of gemcitabine (GEM) and CDDP (GC) has become another standard regimen for advanced UC, because it has been shown to have similar anti-tumoral effects and less toxicity compared with MVAC [4]. However, this regimen has also shown poor outcomes and survival on long-term follow-up, particularly in patients with metastatic UC [3, 5]. Hence, various drugs and regimens have been tried in patients with advanced UC who were refractory to prior chemotherapy. As mentioned above, CDDP is a key agent for first-line chemotherapy. Hence, many investigators have studied use of non-CDDP agents, such as GEM and paclitaxel (PTX), as second- or third-line agents. Previous studies reported minimal toxicity and an overall response rate of approximately 25 % with single-drug therapy with GEM in patients with advanced bladder cancer, while PTX administered as a single agent was reported as producing an overall response rate of 42 % in bladder cancer [6, 7]. Thus, GEM and PTX are regarded as being useful and effective agents for bladder cancer. However, these studies were performed in patients with previously untreated UC. In another report, response rates to single-agent therapy with GEM and PTX were approximately 11 and 7 %, respectively [8, 9]. Based on these facts, many urologists and medical oncologists believe that the efficacy and duration of anticancer effect of GEM and PTX, administered as single agents, are short and insufficient in patients with previous chemotherapy-refractory UC. To overcome this, use of various regimens and schedules combining GEM and PTX therapy (GP therapy) have been reported in advanced UC patients with failure of CDDP-based regimens [10–19]. However, the optimal schedule and dosage of the combination as salvage chemotherapy after failure of CDDP-containing chemotherapy is still controversial.

UC disease progression often results in development of painful and debilitating masses in local and distant organs. In fact, almost all patients with unresectable metastatic and/or locally advanced UC require some kind of analgesic. Although pain is the most devastating symptom of these patients, pain relief is unsatisfactory in some of these patients despite the use of morphine hydrochloride. In addition, analgesics often induce unpleasant side effects and may decrease the patients’ quality of life (QOL). Hence, pain relief with minimal analgesic dosages is an important goal for patients with advanced and recurrent UC. Several reports have demonstrated that GEM and PTX improve pain relief in a variety of cancers [8, 20]. On the other hand, there is little information regarding pain relief with the use of combination GP therapy in patients with advanced UC.

The aim of the present study was to evaluate the anti-tumoral effects and toxicity of low-dose GP (LD-GP) therapy in UC patients with resistance to CDDP-containing therapy. In addition, we paid special attention to pain relief and analgesic consumption in patients receiving this chemotherapy.

## Methods

### Patients

This is a retrospective cohort study of 35 patients, 26 men and nine women, with a median age of 68 years (interquartile range, IQR = 65–77 years), treated with LD-GP therapy for metastatic and/or recurrent UC, previously treated with CDDP-containing chemotherapy at Nagasaki University Hospital from 2003 to 2011. Histologic or cytologic diagnosis of UC was established in all patients. Patients’ characteristics are shown in Table 1. The tumor originated in the upper urinary tract in 13, in the bladder in 22, and in both of them in 1 patient. Eighteen patients had metastasis at the time of initial diagnosis, and 17 patients showed metastasis and/or local recurrence despite previous treatments. All patients received CDDP-based chemotherapy before this therapy and developed progression after undergoing CDDP-based chemotherapy. All patients were required to have a World Health Organization (WHO) performance status (PS) of 0, 1, or 2. The median (IQR) follow-up period was 10 months (4–19 months).

**Table 1:** Patient characteristics

Characteristics	LD-GP therapy (*n* = 35)	Other regimens (*n* = 23)	*P* value
Age, years			0.431
Median/mean	68/69.9	72/67.6	
Interquartile range	65–77	60–75	
Sex (%)			0.159
Male	26 (74.3)	13 (56.5)	
Female	9 (25.7)	10 (43.5)	
Performance status			0.439
0	14 (40.0)	13 (56.5)	
1	16 (45.7)	7 (30.4)	
2	5 (14.3)	3 (13.0)	
Site of primary tumor (%)			0.837
Upper urinary tract	13 (37.1)	10 (43.5)	
Bladder	21 (60.0)	12 (52.2)	
Both	1 (2.9)	1 (4.3)	
Prior treatment (%)			0.110
Chem	8 (22.9)	3 (13.0)	
Chem + Ope	17 (48.6)	12 (52.2)	
Chem + Rad	6 (17.1)	4 (17.4)	
Chem + Ope + Rad	4 (11.4)	4 (17.4)	
Second-/third-line (%)			0.347
Second-line therapy	31 (88.6)	22 (95.7)	
Third-line therapy	4 (11.4)	1 (4.3)	

*LD*-*GP* low-dose combined therapy of gemcitabine and paclitaxel, *Chem* chemotherapy, *OP* operation, *Rad* radiation

As a control group, we evaluated 23 patients who received other treatment regimens (GEM alone, *n* = 7; PTX alone, *n* = 2; PTX + carboplatin, *n* = 9; GEM + carboplatin, *n* = 2; and GEM + CDDP, *n* = 3) during the same period. The clinical features and previous treatments of these patients are shown in Table 1. Although this selection was not randomized, there were no statistical differences in patient characteristics between these two groups. In this study, all 58 patients were diagnosed as UC by histological examination. However, squamous cell carcinoma (SCC) and adenocarcinoma were detected in four and two specimens, respectively. Among these 6 patients, 3 patients with SCC and the 2 patients with adenocarcinoma were treated with LD-GP therapy, while 1 patient with adenocarcinoma was treated with a different therapeutic regimen (GEM + carboplatin).

### Regimen

The GP regimen used in this study was as follows: GEM was administered at a dose of 700 mg/m^2^ intravenously for 30 min on day 1 and 8 of each 28-day cycle. Paclitaxel was administrated at a dose of 70 mg/m^2^ intravenously over 3 h on day 1 and 8 of each 28-day cycle. Dexamethasone sodium phosphate (6.6 mg), diphenhydramine hydrochloride (50 mg), and ranitidine hydrochloride (100 mg) were administered before treatment.

In total, 237 cycles were administered. Thirty-three patients (94.3 %) received at least two cycles of LD-GP therapy. Of the two patients who received less than two cycles, one patient had rapid tumor progression and the other had severe toxicity (leukopenia). Patients received a median of five treatment cycles (IQR = 2–9, range = 1–43). Between 10 and 16 weeks after GP therapy, all patients underwent a computed tomography (CT) scan and/or magnetic resonance imaging (MRI) to determine the in-field tumor response. The local response was assessed using the Response Evaluation Criteria in Solid Tumors guideline version 1.1. [21]. Based on the guidelines, complete response (CR) was defined as the disappearance of all target lesions and reduction of any pathological lymph nodes to <10 mm in the short axis. Partial response (PR) was defined as a decrease in the sum of the longest tumor diameters by at least 30 %. Stable disease (SD) was defined as neither sufficient shrinkage to qualify as PR nor sufficient increase in size to qualify as progressive disease (PD), which was defined as an increase in the sum of the longest tumor diameters by at least 20 %. In addition to the relative increase of 20 %, the sum had to also demonstrate an absolute increase of at least 5 mm. The appearance of new lesion(s) was also considered disease progression.

In this study, almost all (34 of 35, 97.1 %, and 33 of 35, 94.2 %, respectively) of the planned GEM (700 mg/m^2^) and PTX (70 mg/m^2^) doses were administered on day 1 and 8 of each cycle. In two patients with severe toxicities, dosages of GEM and PTX were decreased to 600 mg/m^2^ and 60 mg/m^2^, respectively, ensuring continuation of the therapy. The study protocol was approved by the Institutional Review Board of Nagasaki University Hospital, and all patients provided written informed consent.

### Evaluation of pain relief and adverse events

Since pain is the most important symptom of advanced cancer, the clinical benefit of the treatment was measured by rating pain on a visual analog scale (VAS) of 0–10 (0 indicating no pain and 10 being the most severe pain imaginable). VAS scores were assessed one to 3 days prior to initiating GP therapy and 6–12 weeks after starting the therapy. Positive pain relief was defined as a decrease in analgesic consumption or a decrease in VAS scores without increasing the dose of analgesics. Regulation of analgesic dose, including nonsteroidal anti-inflammatory drugs (NSAIDs) and opioids, was performed by an independent team who were unaware of the study.

Acute toxicities were graded using the Common Toxicity Criteria of the National Cancer Institute (version 3.0). Toxicity was assessed in all patients who received GP.

### Statistical analysis

The primary endpoint of the study was to evaluate pain relief and adverse events after LD-GP therapy. In addition, anti-tumoral effects, including survival rates and duration, were also investigated. Overall survival was measured from the first day of salvage chemotherapy to the day of patient death or last patient contact. Survivals were demonstrated and analyzed using Kaplan–Meier curves and the log-rank *P* test. All patients enrolled in the trial were included in the analyses.

Data are expressed as median (IQR). The Mann–Whitney *U* test was used for analysis of continuous variables. The chi-square test and Fisher’s exact test were used for categorical comparison of the data. All statistical tests were two-sided, and significance was defined as *p* < 0.05. All statistical analyses were performed on a personal computer with the statistical package StatView for Windows (version 5.0, Abacus Concept, Inc., Berkeley, CA).

## Results

### Efficacy

**Fig. 1:**
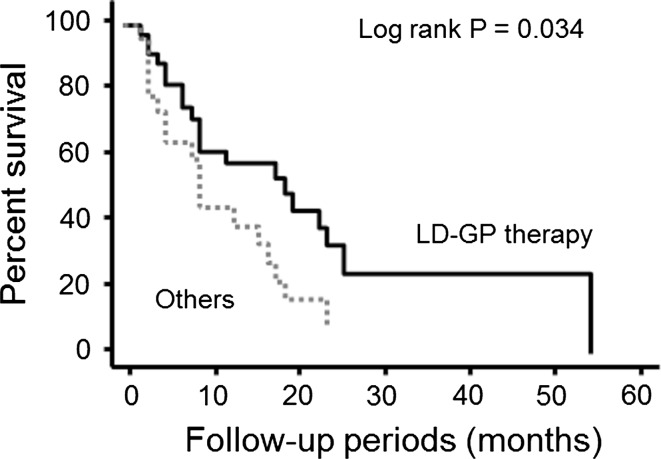
Kaplan–Meier survival curves showed that patients who received LD-GP therapy had a better prognosis compared to those who received other therapeutic regimens (log-rank *p* = 0.034). The other regimens included a combination of paclitaxel and carboplatin, *n* = 9; gemcitabine alone, *n* = 7; combination of gemcitabine and cisplatin, *n* = 3; paclitaxel alone, *n* = 2; and gemcitabine and carboplatin, *n* = 2

The anti-tumor effects of LD-GP therapy and other regimens on measurable solid masses are shown in Table 2. None of the 35 patients who received LD-GP therapy had CR, although nine patients (25.7 %) had PR. PD, on the other hand, was seen in four patients (11.4 %), while 22 patients (62.9 %) had SD. Thus, the major response rate (CR + PR) and disease control rate (CR + PR + SD) were 25.7 % and 88.6 %, respectively. Major response rates and disease control rates in the other regimens group were 17.3 and 73.9 %, respectively. Thus, there were no significant differences in major response rates between the two groups (*p* = 0.220). Kaplan–Meier survival curves are shown in Fig. 1. Median survival rate of patients in the LD-GP therapy group was 12 months (IQR = 6–22 months). With regard to the relationship between survival and PS, median survival (IQR) was 9 months (7–16 months) in patients with a PS of 0, 10 months (6–15 months) in those with a PS of 1, and 5 months (3–12 months) in those with a PS of 2. The 1- and 2-year survival rates after LD-GP therapy were 58.1 and 32.9 %, respectively. On the other hand, median survival and 1- and 2-year survival rates in the other regimens group were 9 months and 44.6 and 8.4 %, respectively. Thus, the survival of patients who received the LD-GP regimen was significantly better than that of those who received other treatment regimens (log-rank *p* = 0.034) (Fig. 1).

**Table 2:** Efficacy of LD-GP and other regimens for measurable tumors

	LD-GP therapy (*n* = 35)	Others regimens (*n* = 23)	*P* value
Complete response	0 (0.0)	1 (4.3)	0.220
Partial response	9 (25.7)	3 (13.0)	
Stable disease	22 (62.9)	13 (56.5)	
Progressive disease	4 (11.4)	6 (26.1)	

*LD*-*GP* low-dose combined therapy of gemcitabine and paclitaxel

### Pain relief

**Fig. 2:**
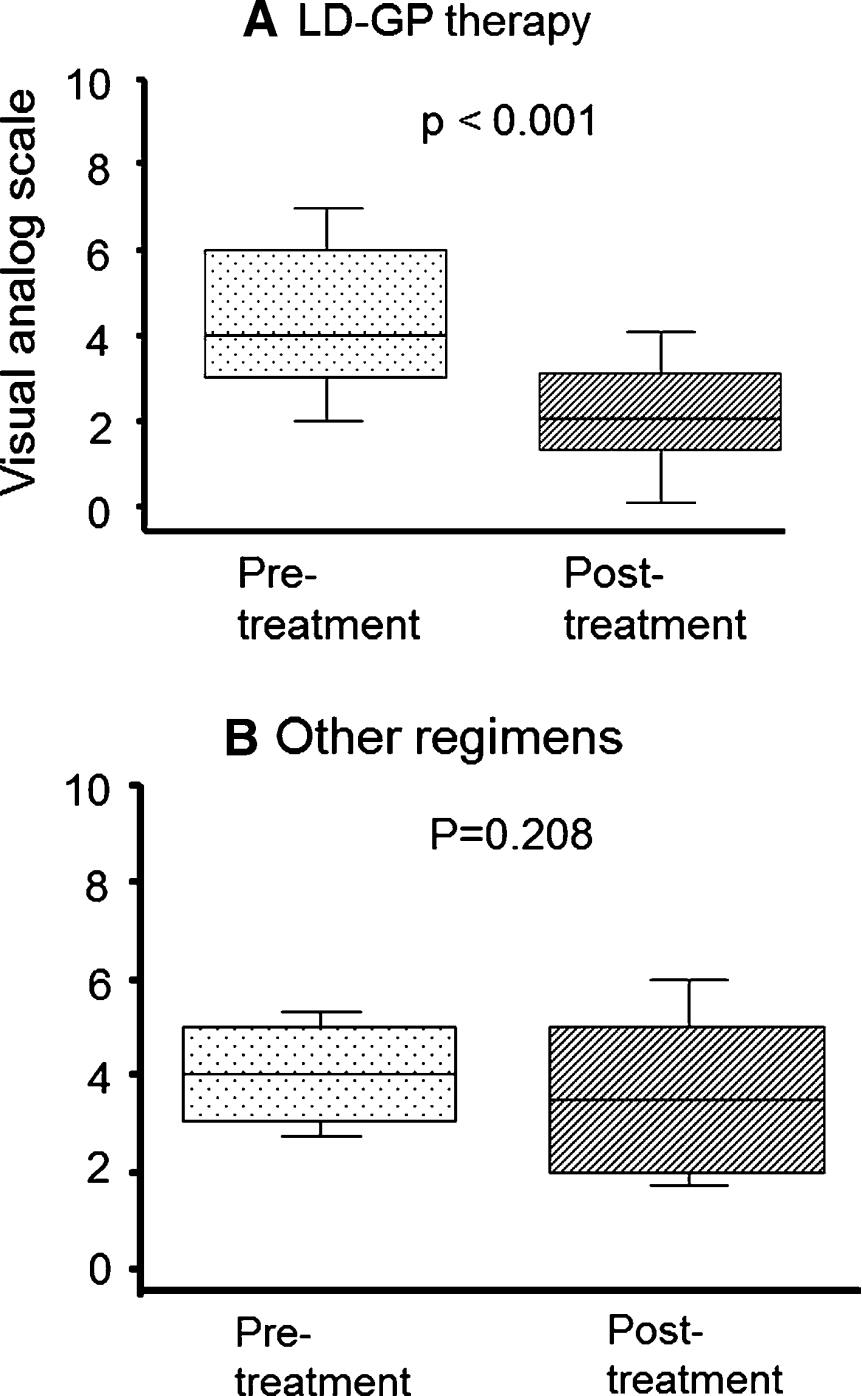
Changes in visual analog scale (VAS) scores after therapy with a low-dose combination of gemcitabine and paclitaxel (LD-GP) (**a**) and other treatment regimens (**b**). VAS scores decreased significantly in the LD-GP therapy group (**a**, *p* < 0.001). Although a similar trend was also found in the other regimens group, the change was not statistically significant (**b**, *p* = 0.208)

The changes in VAS scores with salvage chemotherapy are shown in Fig. 2. At the start of LD-GP therapy, patients complained of abdominal or back pain, with median (IQR) VAS scores of 4 (3–6), due to local and/or metastatic UC. In the other regimens group as well, VAS scores were 4 (3–5), indicating no significant difference between the two groups at the start of treatment (*p* = 0.743). In addition, all patients in both groups needed analgesic agents. After chemotherapy, VAS scores in the LD-GP group and other regimens group were 2 (1–3) and 4 (2–5), respectively, indicating a significant difference in VAS scores after therapy between the two groups (*p* = 0.024). In addition, in the LD-GP group, VAS scores after therapy significantly decreased (*p* < 0.001) compared to their pretreatment levels (Fig. 2a). The change in the other regimens group was, however, not statistically significant (Fig. 2b, *p* = 0.208).

Changes in VAS scores and analgesic requirements after therapy in the two groups are shown in Table 3. Improved pain scores were seen in 24 patients (68.6 %), and decrease in analgesic consumption was seen in 12 patients (34.3 %) in the LD-GP group. Of the 24 patients with improved pain intensity, 8 patients (22.9 %) had improved pain intensity despite decreasing the dose of analgesic. Finally, positive pain relief, that is, a decrease in analgesic consumption or a decrease in VAS scores without increasing the dose of analgesics, was seen in 28 of the 35 patients who received LD-GP therapy (80.0 %). Pain control could not be achieved despite increasing the analgesic dose in only two patients (5.7 %) in the LD-GP group in our study population. On the other hand, in the other regimens group, only seven of 23 patients (30.4 %) were judged as having positive pain relief.

**Table 3:** Changes in visual analog scale scores and analgesic consumption in the two groups

Analgesic consumption in LD-GP therapy		Pain intensity, evaluated by visual analogue scale *n* (%)		
		Improved 24 (68.6 %)	Stable 9 (25.7 %)	Progressed 2 (5.7 %)
Analgesics consumption	*n* (%)			
Decrease	12 (34.3)	8 (22.9 %)	4 (11.4 %)	0 (0.0 %)
No change	21 (60.0)	16 (45.7 %)	5 (14.3 %)	0 (0.0 %)
Increase	2 (5.7)	0 (0.0 %)	0 (0.0 %)	2 (5.7 %)

Analgesic consumption in other regimens group		Pain intensity, evaluated by visual analogue scale *n* (%)		
		Improved 11 (47.8 %)	Stable 13 (56.5 %)	Progressed 3 (13.0 %)
Analgesics consumption	*n* (%)			
Decrease	4 (17.4)	2 (8.7 %)	2 (8.7 %)	0 (0.0 %)
No change	13 (56.5)	3 (13.0 %)	8 (34.8 %)	1 (4.3 %)
Increase	6 (26.1)	6 (26.1 %)	3 (13.0 %)	2 (8.7 %)

*LD*-*GP* low-dose combined therapy of gemcitabine and paclitaxel

### Toxicity

The regimen-related toxicities observed during the study are listed in Table 4. Common myelosuppression-related toxicities included leukopenia and thrombocytopenia, with five patients (14.3 %) requiring treatment with granular colony-stimulating factor (GCSF). In addition, severe thrombocytopenia occurred in two patients (5.7 %), both of whom required platelet transfusions; however, no bleeding episodes occurred.

**Table 4:** Common treatment-related toxicities in the LD-GP group

	Incidence *n* (%)	
	Total	Grade 3 + 4
Myelosuppression-related		
Anemia	6 (17.1)	2 (5.7)
Leukopenia	9 (25.7)	5 (14.3)
Thrombocytopenia	8 (22.9)	2 (5.7)
Non-hemorrhagic complications		
Fatigue	6 (17.1)	0 (0.0)
Nausea/vomiting	4 (11.4)	1 (2.9)
Peripheral neuropathy	4 (11.4)	0 (0.0)
Skin rash	2 (5.7)	1 (2.9)

The most common non-hematologic toxicity in the LD-GP group was fatigue (*n* = 7, 17.1 %), although this was not severe in any of the patients. Severe non-hematologic toxicity was found in two patients (5.7 %). One patient showed severe skin rash, which was, however, deemed by a dermatologist as having no correlation with the treatment regimen, based on professional examinations including a drug-induced lymphocyte stimulation (DLST) test. Although treatment-related pneumonitis was suspected in two patients, they were diagnosed as having cancer-related carcinomatous lymphangiomatosis by a chest physician. None of the patients exhibited a hypersensitivity reaction. In this study, LD-GP therapy had to be discontinued in two patients (5.7 %) because of severe drug-related leukopenia or vomiting. In addition, the GEM and PTX dosages were reduced to 600 and 60 mg/m^2^, respectively, in another two patients (5.7 %) with severe leukopenia. Finally, none of the patients had fatal complications related to the treatment.

## Discussion

CDDP-based combination chemotherapies, such as MVAC and GC therapy, have been extensively studied in patients with advanced UC, with general agreement that they are standard treatments. These therapies improved patient prognosis compared with other single-agent therapy available at the time. Unfortunately, however, these CDDP-based combination chemotherapies were usually not given for long enough to achieve full efficacy, so that the 50 % survival periods were less than 2 years [3]. After 2000, although the GC regimen replaced the MVAC regimen for advanced UC, because of its lower toxicity, its anti-tumoral effect was similar to the MVAC regimen [4]. On the other hand, patients with advanced UC who have recurrent and/or metastatic tumors after first-line therapy inherently have an extremely poor prognosis. Various chemotherapies have been tried as second- or third-line chemotherapy in patients with advanced UC who had received prior cisplatin-based therapy. Patients receiving a combination therapy of PTX, ifosfamide, and nedaplatin showed a high response rate (75 %) and relatively long survival (the 1- and 2-year survival rates were 53.7 and 42.9 %, respectively) [22]. However, all patients who received this therapy had severe (Grade 3 and 4) granulocytopenia, and 25 % of them also had severe thrombocytopenia. In another study, GC therapy was administered as second-line therapy to 33 patients with advanced UC after failure of MVAC therapy [23]. This study showed a response rate of 39.4 % and a 1-year survival rate of 45.6 %. However, this regimen also showed a relatively high frequency of toxicities. Conversely, both GEM and PTX have been reported to be relatively safe and well tolerated in advanced cancer patients. Unfortunately, however, neither of these drugs given as a single agent showed satisfactory efficacy in inhibiting tumor progression and prolonging survival in UC patients with resistance to CDDP-containing therapy. For example, the response rate to a single-agent GEM dose of 1,250 mg/m^2^ given on days 1 and 8 of a 3-week cycle was 11 % and to a single-agent paclitaxel dose of 200 mg on day 1 of a 3-week cycle was approximately 7 % [8, 9]. Many investigators have reported varying responses to various doses and schedules of GP therapy.

Our regimen has several unique differences compared to other GP therapy schedules (Table 5). In our study, doses of GEM and PTX were lowest among all previous reports regarding the use of GP regimens in advanced UC after failure of CDDP-containing chemotherapy. In addition, PTX was administered on day 1 and 8, at the same time as administration of GEM. Various GEM and PTX regimens have been described in previous reports. The most representative and common regimen is administration of GEM on day 1, 8, and 15 and PTX on day 1 [11, 15, 17, 18]. In another report, the day 15 GEM dose was omitted in 31 % of courses, almost always due to myelosuppression [11]. Our previous experience using GP therapy, with 1,000 mg/m^2^ GEM and 150 mg/m^2^ PTX, also showed similar results (data not shown). In addition, it has also been suggested that omission of the day 15 dose may minimize myelosuppression. Hence, in this study, we omitted the day 15 GP dose.

**Table 5:** Previous reports on gemcitabine and paclitaxel therapy after failure of cisplatin-based chemotherapy

Study year (Ref.)	N	Gemcitabine, Paclitaxel (mg/m^2^; day) (every weeks)	CR/PR (%)	SR 1-yr/2-yr (%)	Median survival (mos)	Grade 3/4 leuko-/thrombocytopenia (%)
2001 [10]	41	2,500–3,000; 1 150; 1 (2)	27.5/32.5	NS/NS	14.4	31.7/0.0
2001 [11]	15	1,000; 1, 8, 15 200; 1 (3)	6.7/40.0	NS/NS	NS	NS/NS
2005 [12]	36	1,000; 1, 8, 15 110; 1, 8, 15 (4)	41.7/27.8	NS/NS	15.3	36.1/8.3
2006 [13]	23	2,500; 1 150; 1 (2)	0.0/30.4	NS/NS	12.1	26.1/NS
2006 [14]	14	1,000; 1, 8 175; 1 (3)	50.0/0.0	NS/NS	13	35.7/0.0
	13	1250; 1 120; 2 (2)	7.7/30.8	NS/NS	9	23.1/15.4
2007 [15]	10	1,000; 1, 8, 15 200; 1 (3)	20.0/50.0	40/NS	10.3	50.0/10.0
2008 [16]	20	2,500; 1 150; 1 (2/3)	5.0/25.0	35/NS	11.5	30.0/5.0
2009 [17]	33	1,000; 1, 8, 15 180; 1 (4)	3.0/0.0	NS/NS	11.3	18.2/NS
2011 [18]	24	1,000; 1, 8, 15 200; 1 (3)	8.3/33.3	52/11	12.4	66.7/4.2
2011 [19]	48	1,000; 1, 8 175; 1 (3^a^)	12.5/25.0	NS/NS	7.8	NS/NS
		1000; 1, 8 175; 1 (3^b^)	14.6/26.8	NS/NS	8.0	NS/NS
This study	35	700; 1, 8 70; 1, 8 (4)	0.0/25.7	58.1/32.9	12.0	14.3/8.6

*NS* not shown, *SR* survival rate, *yr* year, *mos* months

^a^A maximum of 6 cycles; ^b^ Given until disease progression

In regard to the dose–response relationship of PTX, one study states that a single application is superior to split doses [14]. In fact, a dose of 80 mg/m^2^ of PTX in a weekly schedule produced only 10 % overall response (CR + PD + SD). Our regimen included a lower dose of PTX (60–70 mg/m^2^). On the other hand, in almost all GP regimens given as salvage chemotherapy, PTX was administered only on day 1. In another regimen in which both GEM (1,000 mg/m^2^) and PTX (110 mg/m^2^) were administered on day 1, 8, and 15, 25 of 36 patients (69.4 %) had a major response to treatment, including 15 patients (41.7 %) with CR [12]. This regimen is reportedly one of the most effective GP regimens described (Table 5). However, patients receiving this regimen were prone to pulmonary toxicity (4 of 24 patients, 16.7 %). Hence, the authors decreased the GEM dose to 800 mg/m^2^ and PTX to 90 mg/m^2^.

Unfortunately, none of the currently available chemotherapeutic agents, including molecular targeted therapy, have proved successful in improving the long-term survival of advanced UC patients after failure of previous chemotherapy. Hence, the most important criteria for salvage chemotherapy for advanced UC are safety, lower drug toxicities, maintenance of patient QOL, and avoidance of hospitalization whenever possible. Keeping this in mind, we planned our current regimen of low-dose combination chemotherapy with GEM and PTX, aiming to prevent disease progression rather than bringing about a cure, with maintenance of QOL. Our regimen was successful in reducing the frequency of severe leukocytopenia and thrombocytopenia, these being less common in our study than with previously used GP regimens. In addition, none of our patients developed severe pulmonary toxicity, neuropathy, or hypersensitivity. Thus, as we expected, LD-GP therapy has the potential to be well tolerated as salvage chemotherapy with minimum adverse events.

At the start of this study, we did not expect a marked response to LD-GP therapy and just hoped to lessen the momentum and velocity of tumor growth and progression. However, although none of the patients in our study had CR with LD-GP and the major response (CR + PR) rate was the lowest (25.7 %) among all previous reports, surprisingly, the median survival was relatively long, beyond our expectations, and longer than the survival times described in other similar reports. For example, the 1-year survival rate in our study was similar to other GP regimens that used high doses of GEM and PTX in advanced UC patients who had previously received platinum-based chemotherapy regimens (57 %) [11]. We are unable to explain the reasons for this favorable phenomenon observed in the present study. However, it is possible that maintenance of QOL and avoidance of the side effects of analgesic drugs may have contributed to the relatively long survival, since nutritional status and physical activity are important effectors of survival in various advanced cancers [24, 25]. In particular, avoidance of severe toxicities may be beneficial in improving the survival in advanced UC patients after failure of previous treatments. From these facts, we speculated that LD-GP therapy affected survival through inhibition of tumor progression and improvement of the patients’ general physical status.

One of the interesting findings of the present study is that LD-GP therapy improved pain relief in advanced UC patients with resistance to CDDP-containing chemotherapy. Actually, 24 of the 35 patients had decreased VAS scores after LD-GP therapy. In addition, a decrease in analgesic consumption was found in approximately one-third of our patients, and a quarter of them had improved pain relief despite a decrease in analgesic consumption. In a previous report on other cancers, three cycles of gemcitabine (1 g/m^2^ on day 1, 8, and 15) produced complete relief of pain in all of the four patients with advanced biliary tract cancer studied [26]. In addition, GEM, administered as a single agent, has been reported to reduce pain in patients with CDDP refractory UC [8]. Weekly paclitaxel also reportedly reduced moderate to severe pain (VAS scores 3–8) from 35.1 to 24.3 % in 37 patients with advanced non-small cell lung cancer [20]. Thus, both GEM and PTX may have some palliative effects on tumor-associated pain. However, we did not observe such phenomena in the other GEM-based and PTX-based regimens administered in this study. Even if these drugs do have significant pain-relieving effects, rapid growth and progression of the tumor may offset them. Furthermore, in the presence of other severe complications, patients are unable to appreciate the pain relief. Based on these facts, we speculated that LD-GP therapy results in an optimal balance between anti-tumoral effects and minimal adverse effects, allowing the patient to appreciate the pain relief.

Further follow-up is necessary to definitively prove the survival benefit of the LD-GP regimen, because the median follow-up period of patients receiving LD-GP therapy in this study was only 10 months and our study population was small. In addition, this study was not a prospective randomized study. Hence, more detailed and larger studies are necessary to decisively conclude about the anti-tumoral effects, including pain control, patient survival, and toxicities of LD-GP therapy. In our opinion, however, LD-GP therapy is feasible and well tolerated as second- or third-line chemotherapy in patients with advanced UC.

## Conclusion

The results of this study demonstrated that LD-GP therapy has anti-tumoral effects in advanced UC patients with resistance to CDDP-containing therapy. In particular, this regimen is useful for pain relief in these patients. The common toxicity associated with the LD-GP therapy in this study was leukopenia, and the most common non-hematologic toxicity was fatigue. However, the frequency and severity of toxicities with the LD-GP regimen used in this study were lower than those with other GP regimes. We speculate that LD-GP therapy is feasible and well tolerated as salvage therapy in patients with advanced UC with resistance to CDDP-containing therapy.
